# Long-Term Outcome of Patients with Stage II and III Muscle-Invasive Urothelial Bladder Cancer after Multimodality Approach. Which Is the Best Option?

**DOI:** 10.3390/medicina59010050

**Published:** 2022-12-27

**Authors:** Oana Gabriela Trifanescu, Laurentia Nicoleta Gales, Mihai Andrei Paun, Natalia Motas, Serban Andrei Marinescu, Ion Virtosu, Raluca Alexandra Trifanescu, Mirela Gherghe, Liviu Bilteanu, Camelia Cristina Diaconu, Rodica Maricela Anghel

**Affiliations:** 1 Department of Oncology, University of Medicine and Pharmacy “Carol Davila”, 050474 Bucharest, Romania; 2 Institute of Oncology “Prof. Dr. Al. Trestioreanu” Bucharest, Radiotherapy II, 022328 Bucharest, Romania; 3 Institute of Oncology “Prof. Dr. Al. Trestioreanu” Bucharest, Medical Oncology II, 022328 Bucharest, Romania; 4 Institute of Oncology “Prof. Dr. Al. Trestioreanu” Bucharest, Thoracic Surgery, 022328 Bucharest, Romania; 5 Department of Thoracic Surgery, University of Medicine and Pharmacy “Carol Davila”, 050474 Bucharest, Romania; 6 Institute of Oncology “Prof. Dr. Al. Trestioreanu” Bucharest, Oncologic Surgery I, 022328 Bucharest, Romania; 7 Institute of Endocrinology “C. I Parhon”, 011863 Bucharest, Romania; 8 Department of Endocrinology, University of Medicine and Pharmacy “Carol Davila”, 050474 Bucharest, Romania; 9 Institute of Oncology “Prof. Dr. Al. Trestioreanu” Bucharest, Nuclear Medicine, 022328 Bucharest, Romania; 10 Department of Nuclear Medicine, University of Medicine and Pharmacy “Carol Davila”, 050474 Bucharest, Romania; 11 Clinical Emergency Hospital of Bucharest, 014461 Bucharest, Romania; 12 Department of Internal Medicine, University of Medicine and Pharmacy “Carol Davila”, 050474 Bucharest, Romania

**Keywords:** bladder cancer, multimodality treatment, chemoradiotherapy, neoadjuvant chemotherapy

## Abstract

*Background and Objectives*: There is no consensus regarding the optimal therapy sequence in stage II and III bladder cancer. The study aimed to evaluate the long-term oncologic outcomes in patients with bladder cancer after a multimodality approach. *Materials and methods*: Medical files of 231 consecutive patients identified with stage II (46.8%), IIIA (30.3%), and IIIB (22.9%) transitional cell carcinoma of the bladder (BC) treated with a multimodality approach were retrospectively reviewed. The treatment consisted of transurethral resections or cystectomy, radiotherapy alone or concurrent chemoradiotherapy as definitive treatment, or neoadjuvant chemotherapy using platinum salt regimens. *Results*: Median age at diagnosis was 65 ± 10.98 years. Radical or partial cystectomy was performed in 88 patients (37.1%), and trans-urethral resection of bladder tumor (TURBT) alone was performed in 143 (61.9%) patients. Overall, 40 patients (17.3%) received neoadjuvant chemotherapy and 82 (35.5%) received definitive chemoradiotherapy. After a median follow-up of 30.6 months (range 3–146 months), the median disease-free survival (DFS) for an entire lot of patients was 32 months, and the percentage of patients without recurrence at 12, 24, and 36 months was 86%, 58%, and 45%, respectively. Patients receiving neoadjuvant chemotherapy had a better oncologic outcome compared to patients without neoadjuvant chemotherapy (median DFS not reached vs. 31 months, *p* = 0.038, HR = 0.55, 95% CI 0.310–0.951). There was a trend for better 3-year DFS with radical cystectomy vs. TURBT (60 months vs. 31 months, *p* = 0.064). Definitive chemoradiotherapy 3-year DFS was 58% compared to 44% in patients who received radiotherapy or chemotherapy alone. *Conclusions*: In patients with stages II and III, both neoadjuvant chemotherapy and concurrent radio-chemotherapy are valid options for treatment and must be part of a multidisciplinary approach.

## 1. Introduction

With a steady-growing incidence worldwide, accounting for 3% of global cancer diagnoses, with an estimated 550,000 people diagnosed in 2018, and with poor prognosis if the disease is diagnosed in an advanced stage (5-year survival rate for metastatic disease is 4.6%), bladder cancer (BC) demands increasing interest regarding the therapeutic management either for curative intent (and undertaking prevention of disease recurrence) or for palliative intent (by attempting to stall the evolution of an incurable disease towards death) [1,2].

BC mainly occurs in the urothelial lining (transitional-cell carcinoma), under constant exposure to the potentially mutagenic agents that get excreted through urine. The risk factors include tobacco smoking [3,4], environmental toxins [4,5], and schistosomiasis infection [6,7]. Hereditary genetic predisposition exists towards developing BC, typically genomic alterations of key enzymes interfering with detoxification of environmental carcinogens (including aromatic amines and tobacco byproducts), such as NAT2 and GSTM1 [8,9,10,11]. Genetic disorders, such as Cowden’s syndrome [12] and Lynch syndrome [1,13,14], can also occur unconnected to any urothelial distress.

Most cases of BC (75%) are localized forms of urothelial cancer (non-muscle invasive bladder cancer—NMIBC) [15] that carry an excellent prognosis, with minimal invasive procedures such as transurethral resections (TURBT), and with the addition of intravesical cytotoxic therapy in high-risk cases, assuring curation [16,17]. The prognosis worsens with underlying smooth muscle involvement (muscle-invasive bladder cancer—MIBC) [1,5,18]. The standard curative option remains surgical resection–radical cystectomy with bilateral lymph node dissection, offering good local control with minimal perioperative mortality [19,20,21,22]. Less radical interventions combine TURBT with chemoradiation and are options for frail patients or for patients who consider a radical intervention as an unacceptable burden to their quality of life [22,23]. Neoadjuvant treatment consisting of induction chemotherapy to downstage the tumor and reduce the incidence of metastasis showed a consistent benefit regarding disease-free survival and 5-year overall survival [22,24,25,26]. All these three categories are listed as category 1 recommendations in the European Guideline [27], thus increasing the uncertainty regarding the best treatment for BC patients.

Beyond surgical treatment, adjuvant treatment is required [21,28,29,30]. The combination of methotrexate, vinblastine, doxorubicin, and cisplatin (MVAC) regimen was the first successful chemotherapy regimen, providing significant improvement compared to its predecessors [31,32,33]. Despite superior outcomes, MVAC was difficult for patients to tolerate, because of substantial toxicities (defined as CTCAE grade ≥ 3), such as neutropenia (including febrile neutropenia), mucositis, renal, cardiac, and neurologic toxicities, and a significant 3–4% death rate [31,32,34]. The gemcitabine–cisplatin (GC) regimen followed showed similar outcomes with a better toxicity profile, leading to the wide adoption of GC as standard first-line adjuvant and metastatic treatment [22,34,35,36,37,38,39]. Intensification of the regimens or addition of new agents have not improved survival; however, dose-dense methotrexate, vinblastine, doxorubicin, and cisplatin (ddMVAC) proved to be better tolerated compared to the standard MVAC regimen [40,41].

The study aimed to evaluate the long-term oncologic outcome of the patients, compare different therapeutic strategies for the treatment of BC, and emphasize the importance of multimodal therapy in BC treatment.

## 2. Materials and Methods

This retrospective study included the medical file data of 231 patients identified with transitional cell carcinoma of the bladder and treated in our oncology institute between 2009 and 2019. All patients submitted their written consent for treatment (chemotherapy, radiotherapy, or both), according to the local policy.

### 2.1. Patient Selection Criteria

All patients included in the analysis have histopathological confirmation of BC and have consecutive admissions to our center for non-surgical treatment (radiotherapy or chemotherapy). The study was approved by the Ethical Committee of the Institute of Oncology (23381/2022). No specific informed consent form (ICF) was used because all patients signed the Institutional ICF giving consent to full use of their medical records for research purposes. The study was conducted in harmonization with the World Medical Association (WMA) Helsinki Declaration of 1975, as revised in 2008.

The prerequisites for treatment criteria were—full recovery post-surgical intervention; ECOG performance status of 0–2; no impeding comorbidities; adequate lab results of the bone marrow reserve (absolute neutrophile count ≥ 1.5 × 10^9^/L, platelet count 100 × 10^9^/L), liver function (bilirubin level ≤ 1.5 mg/dL), and renal function (creatinine level ≤ 1.5 mg/dL); and normal chest X-ray. All patients had cardiology clearance after a consult describing their cardiac status as normal or stable under the necessary treatment for their preexisting cardiovascular disease.

### 2.2. Treatment

The management of BC cases followed a multistep approach. The initial urological examination evaluated the feasibility of curative surgical treatment. Unresectable tumors or inoperable patients underwent transurethral resection of the bladder (TURBT) for tissue sampling and pathology confirmation of urothelial carcinoma. Surgically feasible cases were treated with radical cystectomy, partial cystectomy, or curative TURBT.

The patients were then guided to follow radiation therapy targeting the tumor bed or tumor volume (for surgically and non-surgically treated patients, respectively), 5 days a week one fraction/day, by employing external beam radiotherapy to the bladder and regional pelvic lymph nodes up to a dose of 40 Gy, with a boost to the whole bladder to 54 Gy. The dose could be escalated to a total dose of 64–65 Gy in definitive radiotherapy. Chemotherapy-fit patients received systemic treatment using the following regimens—MVAC every 28 days (Methotrexate 30 mg/m^2^/day on days 1, 14, and 21; Vinblastine 3 mg/m^2^/day on day 2; Adriamycin 30 mg/m^2^/day on day 2; and Cisplatin 70 mg/m^2^/day on day 2) or GC every 21 days (Gemcitabine 1000 mg/m^2^/day on days 1 and 8, plus Cisplatin 70 mg/m^2^/day on day 2).

### 2.3. Statistical Analysis

The analysis centers on determining the disease-free survival (DFS) for patients treated with curative intent (complete resection and adjuvant treatment). Disease-free survival, defined as the time from diagnosis to first recurrence (local or distant) or death by any cause was calculated using the Kaplan–Meier method (performed by SPSS 23.0 for Windows). Metastasis-free survival time (MSF) was defined as the length of time from the start of treatment for cancer that a patient is still alive and the cancer has not spread to other parts of the body. The influence on the survival of relevant parameters was studied by univariate analysis using the log-rank test. Identification of independent prognostic factors was achieved using multivariate analyses performed applying a stepwise Cox proportional hazards model. Significance was considered at the 0.05 level.

## 3. Results

During 2009–2019, a total of 231 consecutive patients with BC received chemotherapy or radiotherapy in our department. The median age at diagnosis was 65 ± 10.98 years old, range of 19–87. Ninety percent of patients were smokers or former smokers. Two-thirds of patients came from urban areas. Hematuria as the first symptom at diagnosis was present in 80.8% of patients (Table 1).

**Figure 1:**
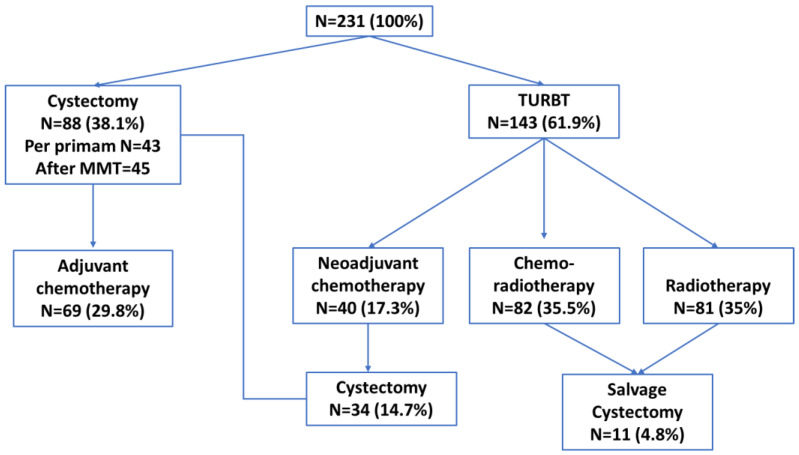
Treatment diagram in the study group. TURBT = trans-urethral resection of bladder tumor, MMT = Multimodality treatment.

Radical or partial cystectomy was performed in 88 patients (38.1%), and trans-urethral resection (TURBT) alone was performed in 143 (61.9%) patients (Figure 1).

Radiotherapy was administered in 81 patients, and concurrent chemoradiotherapy was administered in 82 patients. The mean dose of radiation therapy was 52.4 ± 8.06 Gy and the median was 54 Gy. Radiotherapy was well tolerated, with 84.7% finishing the prescribed dose.

Chemotherapy in neoadjuvant or adjuvant settings was administered in 40 and 69 patients, respectively. Overall, 27 patients received MVAC treatment and 82 received gemcitabine and platinum salt.

**Figure 2:**
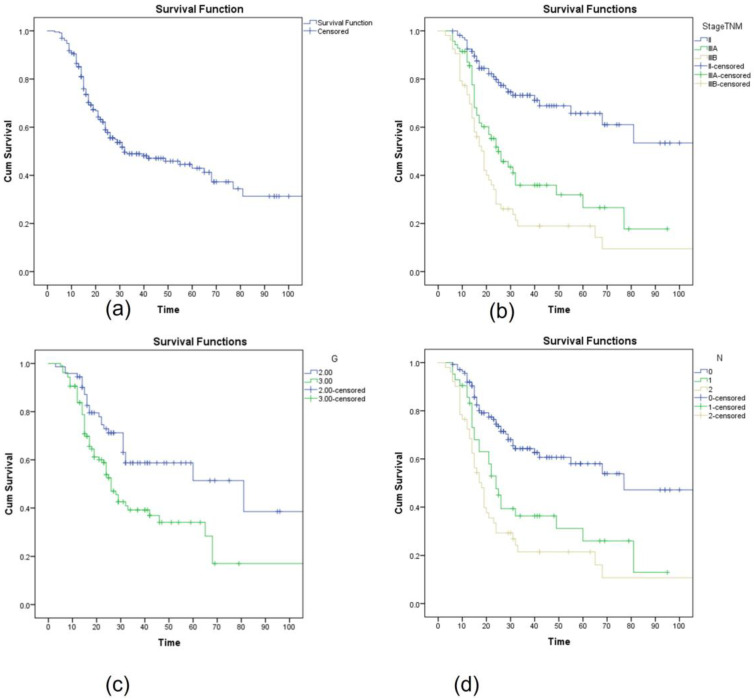
Kaplan–Meier curve assessing the disease-free survival (**a**) for the entire group, (**b**) according to TNM staging, (**c**) according to grading G, and (**d**) according to N status.

After a median follow-up of 30.6 months (range 3–146 months), the median disease-free survival for an entire lot of patients was 32 months, and the percentage of patients without recurrence at 12, 24, and 36 months was 86%, 58%, and 45%, respectively (Figure 2a).

The median disease-free survival according to the stage was as follows: for stage II, median DFS was 120 months; for stage IIIA, it was 25 months; and for stage IIIB, it was only 19 months. (Figure 2b). In patients with T2a tumors, the median DFS was not reached; for T2b, it was at 68 months; for T3a, it was at 32 months; and for T3b, the median DFS was only 16 months. The poorly differentiated G3 tumors had a significantly worse outcome compared to the moderately differentiated tumors (DFS 26 vs. 80 months) (Figure 2c). Patients with N0 disease had an estimated median DFS of 77 months compared to N1 disease 24 months and N2 disease 18 months (Figure 2d).

The relapse patterns were as follows: 19.9% local recurrences, 26.4% distant metastasis, and 4.3% presented with both local recurrence and distant metastasis. The estimated MFS was 68 months. For patients who developed metastasis, the median time to metastasis occurrence was 14 months.

**Figure 3:**
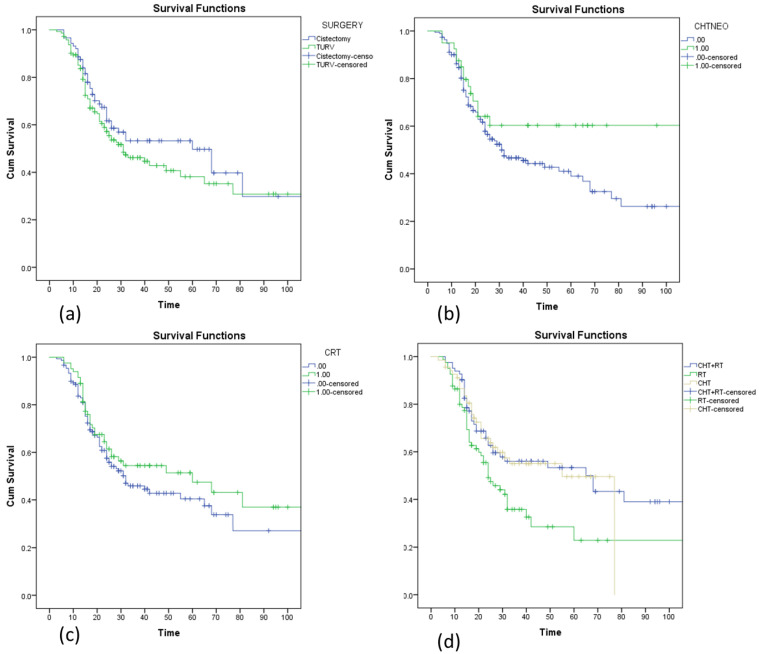
Kaplan–Meier curve assessing the disease-free survival (**a**) in patients who underwent surgery vs. TURBT, (**b**) in patients who underwent definitive chemoradiotherapy (CHT-RT) vs. neoadjuvant chemotherapy (CHT) vs. just radiotherapy (RT), (**c**) in patients who received definitive chemoradiotherapy, and (**d**) in patients who received neoadjuvant chemotherapy (CHTNEO).

Radical cystectomy was associated with a trend of better DFS (60 months vs. 31 months in patients who underwent TURBT), but without reaching statistical significance *p* = 0.064 (Figure 3a).

Forty patients with stage II and stage IIIA disease received neoadjuvant chemotherapy, one of the category 1 recommendation treatments according to guidelines. This percentage is small because an important proportion of patients were cisplatin-ineligible. Patients receiving neoadjuvant chemotherapy had a better outcome, and median DFS was not reached compared to patients without neoadjuvant chemotherapy (31 months vs. not reached *p* = 0.038, HR = 0.55, and 95% CI 0.310–0.951). At 36 months, 48% of the patients without neoadjuvant treatment were free of disease compared to 60% in the group receiving neoadjuvant gemcitabine and cisplatin (Figure 3b). After neoadjuvant chemotherapy, 34/40 patients underwent radical cystectomy.

A vast majority of the patients in our group received TURBT followed by radiotherapy and chemotherapy concurrent or sequential. Concurrent radio-chemotherapy improved DFS interval (60 vs. 32 months) *p* = ns. The percentage of patients who were disease-free after 36 months was 58% for chemoradiotherapy compared to 44% in patients who received just radiotherapy or chemotherapy (Figure 3c).

There is no consensus regarding the optimal therapy sequence in BC. This is reflected in the choice of treatment modality in our patients’ lot. Comparing perioperative treatment in our lot of patients the median estimated DFS was 65 months for chemoradiotherapy, 55 months for neoadjuvant chemotherapy, and only 24 months for patients receiving just RT (Figure 3d).

Neoadjuvant chemotherapy administration improved DFS in patients with stage II from not reached to 81 months; in stage IIIa from 18 to 26 months; and for stage IIIB, the magnitude of benefit was even bigger with an improvement from 17 to 38 months.

Regarding lymph node involvement, chemoradiotherapy was most beneficial in patients with disease localized in the bladder and no lymph node involvement (not reached vs. 26 for N1 disease vs. 18 months for N2 disease), while neoadjuvant chemotherapy strongly improved the prognosis in patients with N1 and N2 disease. In patients who received neoadjuvant chemotherapy with N1 disease, DFS was 81 months vs. 22 months in patients receiving other treatments, and for N2, it was 24 months vs. 18 months.

Receiver Operating Characteristics (ROC) curves were used to measure a possible association between age and the progression of the disease. The Area Under the Curve (AUC) was 0.52, which implies that age does not dictate the oncologic disease-free survival of the patients, and no association was found between age and DFS.

The incidence of side effects (reported in concordance with CTCAE v4.0) during oncologic treatment (radio-chemotherapy and chemotherapy) was significant. The most frequent side effect reported was nausea and vomiting (88.7% of patients in all grades and 17.7 grades 3–4). Febrile neutropenia grade 3–4 was diagnosed in 9.9% of the patients. A decrease in creatinine clearance of less than 30 mL/min/1.73 m^2^ (grade 3 or more) was observed at the end of the treatment in 15.5% of the patients.

## 4. Discussion

Despite the high prevalence of BC, there is no standard treatment to be recommended for stage II and III disease. The treatment must be decided in a multidisciplinary tumor board and should consider the stage of the disease, the performance status, comorbidities, and renal function of the patient; the institution’s facilities; and the patient’s preferences. A multidisciplinary approach including urologists, medical oncologists, and radiation oncologists is necessary [27].

Even in the same tertiary oncology facility, such as our institute, the population of patients with BC was heterogeneous, and the treatment decided in a multidisciplinary tumor board reflects the current guidelines with radical cystectomy for fit patients, neoadjuvant chemotherapy for patients with advanced tumors, and chemoradiotherapy for patients unwilling to undergo radical cystectomy.

Radical surgery treatment of BC involves cystoprostatectomy (including prostate, seminal vesicles, proximal vas deferens, and proximal urethra) in men and cystectomy with hysterectomy in women followed by a urinary diversion [42]. These can be done as open surgery, or (more recently) robotically [43]. Partial cystectomy is feasible in a small proportion of patients, mostly in the dome of the bladder, and is not considered a standard of treatment. The extent of surgery was dictated by the patient’s stage and operability, considering the comorbidities and resectability of the tumor [44,45]. In our lot of patients, only 38.1% of patients underwent radical cystectomy, while in the rest of the patients, only transurethral resection of the bladder was performed. This small percentage was due to patient preference for bladder preservation, comorbidities, and multidisciplinary board decision. In 2022, Zheng et al. evaluated the oncologic outcomes of 22,074 T2 stage MIBC patients, in which only 28% of the patients chose radical cystectomy as the initial treatment, with a vast majority choosing TURBT as the primary surgical treatment. However, the survival rate, OS, and disease-specific survival was significantly better for radical cystectomy [46].

One of the major progresses in the treatment of stage II and IIIA BC is represented by neoadjuvant treatment with platinum salts followed by radical cystectomy [24,25,47]. The advantages of neoadjuvant chemotherapy consist of tumor and lymph node downstaging, micro-metastatic disease eradication, and in vivo assessment of tumor sensibility to cytotoxic agents. A meta-analysis including 11 clinical trials with more than 3000 patients showed an absolute benefit at 5 years regarding an OS of 5% and DFS of 9% (HR 0.86, 95% CI 0.77–0.95) [26]. Another meta-analysis included 15 randomized clinical trials and confirmed an 8% benefit in overall survival in favor of neoadjuvant chemotherapy. The benefit of neoadjuvant chemotherapy was observed only in patients who received cisplatin-based chemotherapy and regarding distant metastasis, not local recurrence [29,48,49]. The most recent meta-analysis found a statistical benefit for patients with T3-T4a, but not for patients with T2a [49].

The proportion of patients receiving cisplatin neoadjuvant chemotherapy in our lot of patients was only 17.3%, due to advanced age, comorbidities, and impaired renal function. Despite strong evidence that supports the use of neoadjuvant chemotherapy, real-world data showed a similar (19%) percent of NACT in stage II and III BC [50]. The absolute benefit in our lot of patients at 3 years regarding DFS was 12%. A recent study showed that even elderly patients (more than 68 years old, but cisplatin eligible) benefit from neoadjuvant chemotherapy, so an increase in the percentage of patients receiving neoadjuvant treatment is expected [51].

The regimens used were MVAC and gemcitabine + cisplatin, similar to those used in clinical trials. A direct comparison between the two regimens, ddMVAC, and GC was conducted in the Vesper phase III trial and reported an advantage regarding response rate, pathologic complete response, and DFS at 3 years in favor of ddMVAC compared to GC, with the expense of higher toxicity [52].

In our group, DFS was statistically better in patients who received neoadjuvant chemotherapy (not reached vs. 31 months). To note: no distant metastasis or local recurrence was recorded after 24 months in the neoadjuvant chemotherapy arm.

Multiple strategies aiming to prevent radical cystectomy without compromising oncologic outcome involved a combination of extended TURBT and radiation therapy or a combination of radiotherapy plus chemotherapy (so-called multimodality or tri-modality treatment). The selection of patients who are candidates for tri-modality treatment includes patients with visible unifocal tumors that are ideally T2, without extensive in situ tumor-associated prognosis, do not invade the prostatic urethra, and are not associated with hydronephrosis, and who have good bladder function and capacity [53].

The results obtained by different retrospective and prospective studies are discordant: one study finds lower cancer-specific survival with multimodality treatment vs. radical cystectomy [54], and some find chemoradiotherapy to be associated with lower 1 to 7 years mortality [55]. A large systematic review found no difference regarding overall survival and DFS between the two treatment modalities [56]. In our lot of patients, it was a clear benefit of chemoradiotherapy compared to radiotherapy alone (median DFS 62 months vs. 24 months in just radiotherapy group).

The big question now is the impact and the necessity of cystectomy (a procedure associated with high mortality, especially in elderly patients) [57] in the era of new treatment options, such as neoadjuvant chemotherapy, immunotherapies, and new more conformational RT techniques [58]. A direct comparison between radical cystectomy and multimodality treatment is difficult in the absence of randomized prospective clinical trials.

In our lot of patients, the difference regarding radical surgery vs. TURBT was not statistically significant even if it was a trend to a better DFS, emphasizing the importance of other treatment modalities, such as neoadjuvant treatment and concurrent chemoradiotherapy. The median disease-free survival for radical surgery, chemoradiotherapy, and neoadjuvant chemotherapy was very close to 5 years.

**Figure 4:**
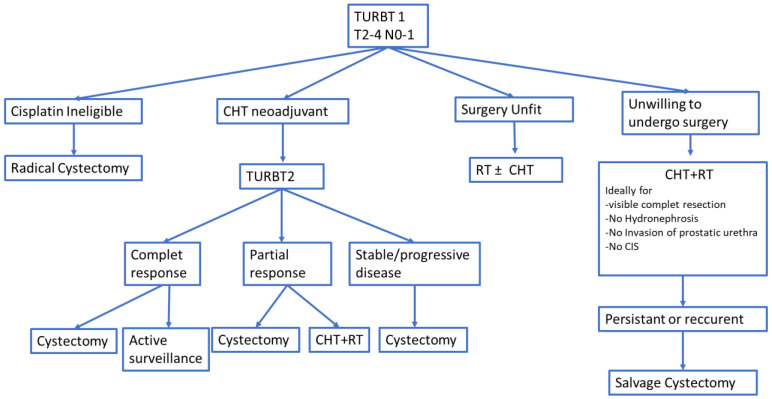
Proposed algorithm for treatment of T_2–4_ N_0–1_ M0 transitional BC (bladder cancer). TURBT = transurethral resection of bladder tumor; CHT = chemotherapy; RT = radiotherapy.

A subgroup analysis of the patients with positive lymph nodes in our study revealed an important benefit from neoadjuvant chemotherapy followed by radical cystectomy. The same conclusion was reported by a clinical study including 639 BC patients with positive lymph nodes showing a 35% reduction in the risk of death if neoadjuvant treatment was administered [59]. An algorithm regarding the treatment of T2-4N0-1 was established to make better treatment decisions in the future (Figure 4).

Correct management according to the latest guidelines of the most common adverse events, such as nausea and vomiting and febrile neutropenia, is mandatory to increase compliance with treatment [60,61].

In the future, based on data reported at the 2021 American Society of Clinical Oncology (ASCO) Annual Meeting, immunotherapy will be incorporated in bladder preservation protocols, showing a good response rate for pembrolizumab, nivolumab, and durvalumab, but data are still immature [62,63,64]. At the time of this study, immunotherapy is not approved for adjuvant treatment of BC.

## 5. Conclusions

Treatment decisions and the sequence of patients with BC stage II and III must be decided in an experimented tumor decision board and involve multiple treatment modalities. Neoadjuvant chemotherapy and chemoradiotherapy clinically significantly improved the oncologic outcome of the patients. Radiotherapy and cystectomy remain the most important treatment modality in cisplatin-ineligible patients.

## Figures and Tables

**Table 1:** Patients’ demographic characteristics.

Patients’ Characteristics	(n = 231)
Female/Male	41/190 (17.75/82.25%)
Urban/rural area	156/84 (65/35%)
Age	65 ± 10.98
Staging II/IIIA/IIIB	108/70/53 (46.8%/30.3%/22.9%)
T2a/T2b/T3a/T4a	25.9%/27.7%/19.5%/19.1%/7.8%
N0/N1/N2	59.8%/18.2%/22%
G2/G3	40.3%/59.7%
Surgery (Cystectomy vs. TURBT)	88/143 (38.1%/61.9%)
Neoadjuvant Chemotherapy	40 (17.3%)
Chemotherapy adjuvant	69 (29.8%)
Chemoradiotherapy	82 (35.5%)
Radiotherapy	81 (35%)

## Data Availability

Not applicable.
